# Supplementary material for: Utility of a Multimodal Biomarker Panel and Serum Proapoptotic Activity to Refine Diagnosis of Ovarian Adnexal Masses
Source: Diseases. 2025 Oct 16;13(10):342. doi: 10.3390/diseases13100342 (PMC12563309; doi:10.3390/diseases13100342)
Supplement: Supplementary file 1 [file diseases-13-00342-s001.zip › diseases-3836192-supplementary.pdf]

**Supplementary Table S1**. Analysis of feature distribution and statistical comparisons between patient groups.

| Feature | Control (n=9) | Benign (n=87) | Malignant (n=40) | KW p-value | Effect Size ($\eta^2$) | Control vs Benign | Control vs Malignant | Benign vs Malignant |
|---|---|---|---|---|---|---|---|---|
| Apoptosis | 65.0 +/- 23.7, 69.9 [54.4-81.6] | 28.8 +/- 21.6, 22.4 [13.5-36.5] | 18.8 +/- 15.1, 12.3 [8.5-24.4] | < 0.001*** | 0.164 | < 0.001*** | < 0.001*** | 0.006** |
| CA-125 | 18.8 +/- 8.8, 19.3 [16.3-22.8] | 129.6 +/- 176.5, 50.7 [17.8-178.7] | 478.6 +/- 294.9, 549.9 [249.4-736.5] | < 0.001*** | 0.311 | 0.013* | < 0.001*** | < 0.001*** |
| CCL11 | 14.2 +/- 8.2, 14.9 [7.6-19.0] | 19.5 +/- 17.1, 16.7 [6.2-27.5] | 25.4 +/- 22.6, 21.3 [7.1-36.0] | 0.371 | 0.000 | NS | NS | NS |
| CCL20 | 4.03 +/- 1.78, 3.59 [2.75-5.22] | 8.92 +/- 18.09, 3.55 [2.27-5.98] | 13.9 +/- 31.3, 4.93 [2.50-11.01] | 0.367 | 0.000 | NS | NS | NS |
| CXCL1 | 47.9 +/- 25.3, 46.5 [29.9-65.4] | 55.6 +/- 52.7, 44.8 [22.2-71.7] | 90.4 +/- 86.6, 65.4 [24.1-149.9] | 0.161 | 0.013 | NS | NS | NS |
| CXCL10 | 62.9 +/- 51.8, 55.5 [22.8-74.5] | 74.3 +/- 52.7, 61.3 [41.4-91.2] | 88.1 +/- 63.8, 68.8 [40.7-113.5] | 0.457 | 0.000 | NS | NS | NS |
| CXCL2 | 376.8 +/- 244.9, 365.0 [154.5-450.0] | 548.3 +/- 489.0, 453.2 [233.1-765.2] | 687.8 +/- 492.5, 520.9 [382.2-971.0] | 0.073 | 0.026 | NS | NS | NS |
| CXCL9 | 99.8 +/- 110.0, 51.5 [28.7-144.4] | 140.3 +/- 141.9, 93.9 [41.7-169.1] | 309.1 +/- 691.6, 108.0 [46.6-196.5] | 0.246 | 0.006 | NS | NS | NS |
| Cystatin C | 4589 +/- 2898, 4915 [3248-5824] | 4179 +/- 2544, 3767 [3153-4727] | 5202 +/- 2205, 4500 [3606-6221] | 0.103 | 0.031 | NS | NS | 0.031* |
| G-CSF | 14.5 +/- 29.1, 0.00 [0.00-2.07] | 96.4 +/- 132.5, 51.3 [0.0-130.6] | 80.0 +/- 93.2, 64.1 [0.0-113.8] | 0.041* | 0.035 | 0.015* | 0.013* | NS |
| HE4 | 2149 +/- 580, 1987 [1902-2293] | 2937 +/- 1252, 2742 [2020-3529] | 4479 +/- 1810, 4913 [3814-5438] | < 0.001*** | 0.201 | 0.038* | < 0.001*** | < 0.001*** |
| ICAM-1 | 361.3 +/- 319.5, 294.1 [159.6-537.8] | 277.3 +/- 171.9, 237.1 [177.4-386.9] | 345.2 +/- 189.6, 301.9 [216.7-431.2] | 0.206 | 0.014 | NS | NS | NS |
| IGFBP-4 | 1938 +/- 1063, 2095 [1602-2664] | 1505 +/- 842, 1313 [1014-1812] | 2206 +/- 1070, 2128 [1588-2470] | 0.002** | 0.129 | NS | NS | < 0.001*** |
| IL-6 | 4.54 +/- 1.79, 4.83 [4.49-5.34] | 42.0 +/- 114.9, 7.65 [5.25-26.81] | 28.8 +/- 44.5, 11.7 [7.1-28.7] | < 0.001*** | 0.098 | 0.006** | < 0.001*** | NS |

| | | | | | | | | |
|---|---|---|---|---|---|---|---|---|
| IL-8 | 1.98 +/- 2.96,<br>0.00 [0.00-5.86] | 8.79 +/- 19.38,<br>0.00 [0.00-7.18] | 20.2 +/- 29.8,<br>7.91 [0.48-33.58] | 0.001** | 0.092 | NS | 0.015* | < 0.001*** |
| MMP-2 | 1592 +/- 1146,<br>1512 [973-2452] | 1355 +/- 4840,<br>459.1 [0.1-987.1] | 813.3 +/- 752.6,<br>742.5 [0.1-1142.6] | 0.026* | 0.064 | 0.010* | 0.047* | NS |
| MMP-9 | 4552 +/- 2782,<br>4870 [3381-6083] | 2983 +/- 1699,<br>2813 [1911-3572] | 3810 +/- 2052,<br>3251 [2469-4872] | 0.077 | 0.038 | 0.048* | NS | NS |
| MPO | 2630 +/- 1358,<br>2300 [2138-3253] | 2540 +/- 1950,<br>2083 [1313-3603] | 2945 +/- 1935,<br>2650 [1706-3244] | 0.378 | 0.000 | NS | NS | NS |
| MRP8/14 | 39888 +/- 29237,<br>38949 [26337-43838] | 139958 +/- 501686,<br>40819 [24328-74595] | 383704 +/- 835116,<br>104025 [51317-191526] | < 0.001*** | 0.245 | NS | < 0.001*** | < 0.001*** |
| Myoglobin | 672.1 +/- 585.4,<br>532.3 [368.3-626.1] | 679.4 +/- 669.9,<br>471.5 [286.9-848.8] | 834.8 +/- 1308.5,<br>440.1 [320.0-842.3] | 0.995 | 0.000 | NS | NS | NS |
| NGAL | 1988 +/- 1281,<br>2086 [1388-2553] | 1936 +/- 1407,<br>1633 [1009-2410] | 2059 +/- 1425,<br>1721 [1309-2438] | 0.736 | 0.000 | NS | NS | NS |
| OPN | 297.8 +/- 153.6,<br>355.8 [172.7-415.3] | 412.1 +/- 521.3,<br>319.7 [153.3-493.2] | 2003 +/- 8126,<br>600.4 [327.7-828.0] | 0.002** | 0.132 | NS | 0.007** | 0.002** |
| ROMA | 26.3 +/- 14.9,<br>20.4 [14.8-34.0] | 35.7 +/- 20.7,<br>30.7 [19.8-48.6] | 67.5 +/- 26.8,<br>78.9 [53.5-90.5] | < 0.001*** | 0.249 | NS | < 0.001*** | < 0.001*** |
| SAA | 12567 +/- 13730,<br>8250 [4283-10706] | 15547 +/- 15262,<br>9226 [3723-22339] | 31152 +/- 24983,<br>26958 [12122-42791] | 0.002** | 0.134 | NS | 0.009** | 0.002** |
| sFAS | 221.9 +/- 140.5,<br>195.4 [146.8-229.7] | 470.3 +/- 679.8,<br>278.2 [149.7-461.3] | 377.3 +/- 377.1,<br>264.2 [101.8-495.2] | 0.458 | 0.000 | NS | NS | NS |
| VCAM-1 | 13154 +/- 9040,<br>14460 [4367-20476] | 11800 +/- 5979,<br>11253 [9189-13829] | 12560 +/- 6270,<br>10982 [9570-12268] | 0.874 | 0.000 | NS | NS | NS |

Descriptive and comparative statistics are presented for the 26 features included in the analysis: 24 soluble molecules, 1 predictive index (ROMA), and 1 independent death-induction assay (Apoptosis). Data are presented as mean +/- standard deviation and median [Q1-Q3 interquartile range]. Statistical comparisons between groups were performed using the Kruskal-Wallis test (KW). Pairwise comparisons were conducted using the Mann-Whitney U test. The effect size for the KW test is reported as eta-squared ($\eta^2$), interpreted as follows: small ($\eta^2 \geq 0.01$), medium ($\eta^2 \geq 0.06$), and large ($\eta^2 \geq 0.14$). Significance levels are denoted as *$p < 0.05$, **$p < 0.01$, ***$p < 0.001$; NS indicates not significant ($p \geq 0.05$).
